# Perceived Stigma and Mental Health Disorders Among Adults With Alopecia Areata Living in Japan

**DOI:** 10.1111/1346-8138.17831

**Published:** 2025-06-27

**Authors:** Norimitsu Saito, Kazumasa Kamei, Genevieve Gautier, Samantha K. Kurosky, Kent A. Hanson, Griffith Bell, Lulu Lee, Nikoletta Sternbach, Akira Yuasa

**Affiliations:** ^1^ Nagomi Dermatology Clinic Kanagawa Japan; ^2^ Pfizer Japan Inc. Tokyo Japan; ^3^ Pfizer Inc. Kirkland Quebec Canada; ^4^ Pfizer Inc. New York New York USA; ^5^ Department of Pharmacy Systems, Outcomes and Policy University of Illinois Chicago Chicago Illinois USA; ^6^ Oracle Life Sciences Austin Texas USA

**Keywords:** alopecia areata, cross‐sectional studies, Japan, mental health, shame

## Abstract

Alopecia areata (AA) is a common disorder that causes hair loss and can significantly impact quality of life, which may be partially due to AA‐related stigma. Examining the impact of AA on psychosocial health is important for understanding the burden experienced by patients with AA. The primary objective of this study was to examine mental health and sleep conditions, hair growth satisfaction, and AA‐related stigma perceptions among individuals diagnosed with AA in Japan. The study used patients' self‐reported data collected from the National Health and Wellness Survey conducted in Japan in 2023. Collected data included demographic characteristics and comorbidities; among those with a self‐reported clinical diagnosis of AA, additional information on clinical characteristics, treatments, and perceived AA‐related stigma was captured. Results were analyzed and stratified by self‐assessed disease severity. Among the full sample (30 013 adults living in Japan), 471 respondents reported a clinical diagnosis of AA, including 347 mild cases, 100 moderate cases, and 24 severe cases. A diagnosed mental health disorder in the past year was reported by 57 respondents (12.1%), and 67 (14.2%) reported a diagnosed sleep condition in the past year. Less than half of respondents (47.4%) were satisfied with their current hair growth, and satisfaction decreased with increasing disease severity. Overall, 70.3% of respondents reported feelings of embarrassment, 55.0% felt that others judged them negatively, and 50.3% felt that others treated them negatively due to AA. A higher proportion of respondents with a severe case (54.2%) reported feeling embarrassed to have AA “very much so” compared with respondents who had mild (15.3%) or moderate (26.0%) cases. Perceived AA‐associated stigma increased with disease severity. Overall, this study demonstrated the prevalence of AA‐related disease stigma and mental health conditions among individuals with AA living in Japan, underscoring the importance of mental health support for patients with AA.

## 1 Introduction

Alopecia areata (AA) is an autoimmune disease that has an underlying immuno‐inflammatory pathogenesis and is characterized by nonscarring hair loss of the scalp, face, and/or body [1]. The global prevalence of AA is approximately 0.58%–2% [2, 3, 4, 5, 6]. Prevalence of AA in Japan varies depending on study methodology. An analysis of a web‐based survey conducted in 2021 estimated the self‐reported population prevalence rate of AA to be approximately 1.45% in Japan [7], whereas an analysis of a claims database showed that the prevalence of diagnosed AA in 2019 was 0.22%–0.33% [8]. These results suggest that there are many patients with AA who do not see a specialist.

AA can significantly impair quality of life [9, 10, 11]. Individuals with AA report AA‐related psychosocial effects, including negative impacts on relationships and self‐esteem [7, 12]. Patients with AA can also experience a range of mental health conditions, most commonly anxiety and depression [6, 10, 11, 12, 13]. In addition, patients with AA in Japan have demonstrated low productivity at work (presenteeism), which has contributed to a nationwide and personal economic burden [14].

Examining the impact of AA on mental and psychosocial health may lead to a better understanding of the unmet needs and burdens faced by patients with AA. Stuhlmann et al. (2024) conducted a systematic review of self‐stigma among individuals who had one of five chronic skin diseases, including AA [15]. Out of the 27 studies identified in the systematic review, only three included patients with AA, which were conducted in Japan [16], Austria [17], and Turkey [18]. The main objective of the Japanese study was to evaluate the reliability and validity of the Japanese version of the Cutaneous Body Image Scale (CBIS); however, a limited number of patients with AA were included (7.5% of 165 dermatology patients) [16]. To our knowledge, quantitative evidence of AA‐related stigma is limited not only worldwide but also in Japan.

The primary objective of this study was to examine mental health and sleep conditions, satisfaction level with hair growth, and perceptions of AA‐related stigma among a sample of individuals diagnosed with AA living in Japan. Furthermore, this study sought to describe the sociodemographic characteristics, comorbidity profiles, and treatment experiences of the study population.

## 2 Methods

### 2.1 Study Design

This study used patient‐reported data collected in 2023 from the Japan National Health and Wellness Survey (NHWS), an annual, self‐reported, syndicated, cross‐sectional, general population survey [19]. Data collection and recruitment primarily occurred online between June and August 2023. Respondents aged ≥ 18 years and living in Japan were recruited from opt‐in survey panels from Profiles and its partners and were invited to complete the survey via email invitation. All respondents were required to provide consent to be eligible for the study and received points as compensation upon completing the study. Respondents were recruited via a quota sampling approach to mirror the general adult population distribution for age and sex in Japan. Respondents completed a base survey that included questions on demographics, personal health history, resource utilization, and attitudes and behaviors on general health. Among those who self‐reported experiencing AA in the base survey, an AA‐specific module, which included additional questions related to AA, was also completed. Collected data included self‐reported demographics, health conditions, AA‐specific clinical characteristics and treatments, and perceived AA‐specific stigma. Adults aged ≥ 18 years with a self‐reported clinical diagnosis of AA were included in the study. The Pearl Institutional Review Board (Indianapolis, IN) reviewed and granted exemption status for Japan 2023 NHWS.

### 2.2 Outcomes

Prevalence of self‐reported clinical diagnosis of AA was calculated from the full survey population. Respondents' demographic characteristics, AA clinical characteristics, AA treatment, mental health and sleep disorders, and perceived AA‐related stigma were analyzed and stratified across self‐assessed disease severity (mild, moderate, and severe). AA disease severity was self‐reported by respondents and based on their own assessment of severity. Disease severity was not reported or confirmed by a physician. In addition, the Scalp Hair Assessment patient‐reported outcome (SHA PRO) measure was used to assess the extent of current scalp hair loss [20]. The survey included questions on perceived AA‐associated stigma to assess experiences of AA‐related stigma among respondents. The following questions were included: (1) Do you feel embarrassed to have alopecia areata? (2) Do you think that others judge you negatively if they know you have alopecia areata? and (3) Do you think that others treat you negatively if they know you have alopecia areata? Respondents answered these questions related to stigma on a scale of 1–7, in which 1 indicated no feelings at all and 7 indicated very strong feelings.

### 2.3 Statistical Analysis

Results were primarily reported as unweighted percentages. Prevalence data were weighted based on age and sex to match the demographic composition of the adult population (age ≥ 18 years) in Japan using the International Database of the US Census Bureau and Organisation for Economic Co‐operation and Development [21]. Categorical variables were reported as frequencies and percentages, and continuous and discrete variables as means and standard deviations (SDs). All analyses were performed using Unicom Systems Inc., Unix Quantum Version 5.8.1 (Mission Hills, CA).

## 3 Results

### 3.1 Respondents

**FIGURE 1:**
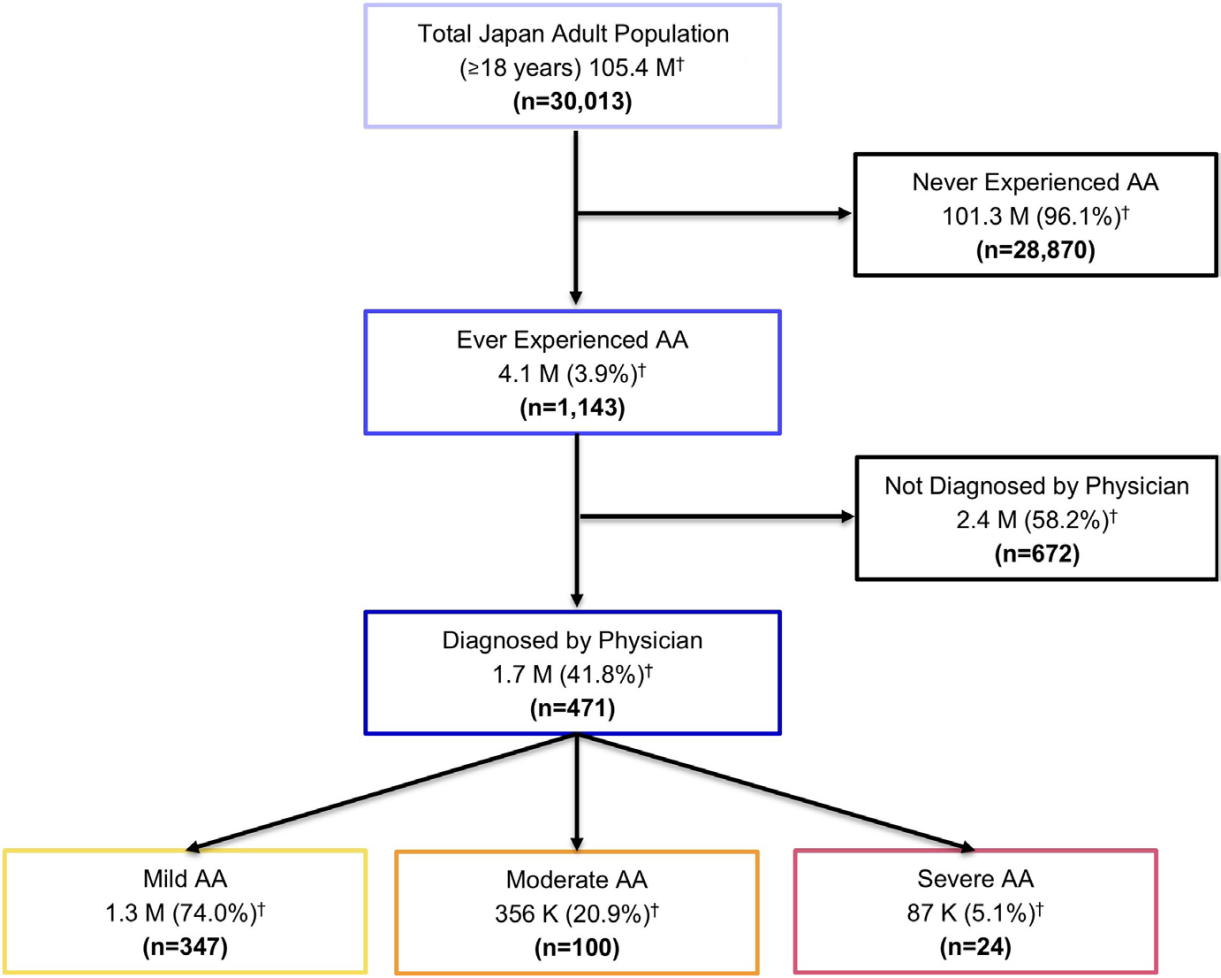
Responder flow diagram. AA, alopecia areata; K, thousand; M, million. ^†^Weighted to the demographic distribution of Japan.

A sample of 30 013 adults (mean age [SD], 52.6 [17.1] years; weighted *n* = 105.4 million) completed the base survey, with 1143 (weighted 3.9%, *n* = 4.1 million) reporting ever experiencing AA and 471 (weighted 1.6%, *n* = 1.7 million) reporting a clinical diagnosis of AA (Figure 1). Among those with a self‐reported clinical diagnosis of AA (*n* = 471), 347, 100, and 24 respondents self‐reported a mild, moderate, and severe case, respectively (Table 1). The mean (SD) age of respondents diagnosed with AA was 56.5 (15.0) years, with 50.5% of respondents ≥ 60 years old, and 59.9% were female.

**TABLE 1:** Demographics and baseline characteristics by self‐reported disease severity^a^.

Characteristic	Diagnosed with AA			
	Total (*N* = 471)	Mild^a^ (*n* = 347)	Moderate^a^ (*n* = 100)	Severe^a^ (*n* = 24)
Age				
Mean (SD)	56.5 (15.0)	57.2 (15.0)	56.4 (14.8)	47.6 (14.0)
Median (Q1, Q3)	60.0 (47.0, 68.0)	61.0 (47.0, 69.0)	58.5 (47.0, 68.0)	49.5 (36.5, 58.5)
Sex, *n* (%)				
Female	282 (59.9)	208 (59.9)	60 (60.0)	14 (58.3)
Male	189 (40.1)	139 (40.1)	40 (40.0)	10 (41.7)
Employment status, *n* (%)				
Full time, part time, or self‐employed	250 (53.1)	182 (52.5)	51 (51.0)	17 (70.8)
Unemployed^b^	221 (46.9)	165 (47.6)	49 (49.0)	7 (29.2)
Insurance coverage, *n* (%)				
National health insurance	221 (46.9)	165 (47.6)	46 (46.0)	10 (41.7)
Social insurance	192 (40.8)	141 (40.6)	40 (40.0)	11 (45.8)
Late‐stage elderly insurance	32 (6.8)	24 (6.9)	8 (8.0)	—
Other	17 (3.6)	15 (4.3)	2 (2.0)	—
None of the above	9 (1.9)	2 (0.6)	4 (4.0)	3 (12.5)
Diagnosed comorbidities, *n* (%)				
Atopic dermatitis (in the past 12 months)	46 (9.8)	33 (9.5)	4 (4.0)	9 (37.5)
Thyroid condition (ever)	32 (6.8)	24 (6.9)	6 (6.0)	2 (8.3)
Psoriasis (ever)	18 (3.8)	15 (4.3)	2 (2.0)	1 (4.2)
Rheumatoid arthritis (ever)	15 (3.2)	13 (3.8)	1 (1.0)	1 (4.2)
Psoriatic arthritis (ever)	5 (1.1)	4 (1.2)	—	1 (4.2)
Vitiligo (ever)	3 (0.6)	2 (0.6)	—	1 (4.2)
Type 1 diabetes (ever)	2 (0.4)	1 (0.3)	—	1 (4.2)
Emotional or mental health conditions in the past 12 months^c^	57 (12.1)	41 (11.8)	10 (10.0)	6 (25.0)
Depression	41 (8.7)	30 (8.7)	8 (8.0)	3 (12.5)
Panic disorder	18 (3.8)	11 (3.2)	3 (3.0)	4 (16.7)
Anxiety	14 (3.0)	11 (3.2)	1 (1.0)	2 (8.3)
Sleep conditions in the past 12 months^d^	67 (14.2)	53 (15.3)	9 (9.0)	5 (20.8)
Insomnia	55 (11.7)	42 (12.1)	9 (9.0)	4 (16.7)
Sleep apnea	13 (2.8)	11 (3.2)	—	2 (8.3)
Narcolepsy	2 (0.4)	1 (0.3)	—	1 (4.2)
Sleep difficulties^e^	6 (1.3)	4 (1.2)	—	2 (8.3)

Abbreviations: AA, alopecia areata; SD, standard deviation.

^a^ AA disease severity (mild, moderate, or severe) was self‐reported by respondents and based on their own assessment of severity.

^b^ Includes homemaker, retired, student, short‐ or long‐term leave of absence due to illness, not employed but looking for work, and not employed and not looking for work.

^c^ Other selected conditions included attention deficit/deficit and hyperactivity disorder, generalized anxiety disorder, obsessive compulsive disorder, phobias, post‐traumatic stress disorder, and social anxiety disorder. Anxiety, depression, and panic disorder are not mutually exclusive.

^d^ Other selected conditions included idiopathic hypersomnia.

^e^ Other than insomnia, narcolepsy, or sleep apnea.

Overall, 12.1% (*n* = 57) of respondents diagnosed with AA reported experiencing a diagnosed emotional or mental health condition in the past year, and 14.2% (*n* = 67) experienced a diagnosed sleep condition in the past year (Table 1), whereas, among the 30 013 adults surveyed, 5.6% (*n* = 1668) reported experiencing a diagnosed emotional or mental health condition in the past year, and 5.1% (*n* = 1544) experienced a diagnosed sleep condition in the past year (results not reported). Higher proportions of respondents with a severe case reported a diagnosed mental health disorder (25.0%) and a diagnosed sleep disorder (20.8%) in the past year compared with those reporting a mild (11.8% and 15.3%, respectively) or moderate (10.0% and 9.0%, respectively) case.

Most individuals (77.1%) were diagnosed with AA by a dermatologist, and the mean (SD) years since diagnosis was 21.3 (16.7) years (Table 2). The proportions of respondents self‐reporting current scalp hair loss of 0%, 1%–20%, 21%–49%, and ≥ 50% were 29.3%, 59.5%, 6.2%, and 5.1%, respectively, per the SHA PRO. Most respondents self‐reported having mild (73.7%; *n* = 347) or moderate (21.2%; *n* = 100) cases. Demographics, baseline characteristics, and clinical characteristics by respondent scalp hair loss range are shown in Table S1.

**TABLE 2:** Clinical characteristics of respondents with AA by self‐reported disease severity^a^.

Characteristic	Diagnosed with AA			
	Total (*N* = 471)	Mild^a^ (*n* = 347)	Moderate^a^ (*n* = 100)	Severe^a^ (*n* = 24)
Diagnosing physician for AA, *n* (%)				
Dermatologist	363 (77.1)	264 (76.1)	85 (85.0)	14 (58.3)
General internist	61 (13.0)	45 (13.0)	10 (10.0)	6 (25.0)
Other	35 (7.4)	28 (8.1)	4 (4.0)	3 (12.5)
Pediatrician	8 (1.7)	6 (1.7)	1 (1.0)	1 (4.2)
Unknown/not reported	4 (0.9)	4 (1.2)	—	—
Time since first diagnosis, mean (SD), years	21.3 (16.7)	21.7 (16.8)	19.1 (16.1)	23.8 (17.9)
Scalp Hair Assessment PRO, *n* (%) [20]				
0% of scalp hair loss^b^	138 (29.3)	118 (34.0)	18 (18.0)	2 (8.3)
1%–20% of scalp hair loss^b^	280 (59.5)	214 (61.7)	61 (61.0)	5 (20.8)
21%–49% of scalp hair loss^b^	29 (6.2)	13 (3.8)	14 (14.0)	2 (8.3)
50%–94% of scalp hair loss^b^	11 (2.3)	1 (0.3)	3 (3.0)	7 (29.2)
95%–100% of scalp hair loss^b^	13 (2.8)	1 (0.3)	4 (4.0)	8 (33.3)
Currently receiving treatment for AA, *n* (%)^c^				
Yes	38 (8.1)	24 (6.9)	10 (10.0)	4 (16.7)
Currently receiving phototherapy, *n* (%)				
Yes	26 (5.5)	13 (3.8)	8 (8.0)	5 (20.8)
Approaches currently using to address AA, *n* (%)				
Headwear^d^	46 (9.8)	21 (6.1)	16 (16.0)	9 (37.5)
Wigs	14 (3.0)	2 (0.6)	5 (5.0)	7 (29.2)
Tattoos mimicking the appearance of hair^e^	2 (0.4)	—	—	2 (8.3)
Hair styling practices^f^	19 (4.0)	10 (2.9)	6 (6.0)	3 (12.5)
Psychological counseling for anxiety, depression, or sleep disorder related to AA	5 (1.1)	3 (0.9)	—	2 (8.3)
Prescribed medication for treatment of anxiety, depression, or sleep disorder related to AA	7 (1.5)	2 (0.6)	3 (3.0)	2 (8.3)
Other	9 (1.9)	5 (1.4)	2 (2.0)	2 (8.3)
None of the above	397 (84.3)	309 (89.1)	76 (76.0)	12 (50.0)

Abbreviations: AA, alopecia areata; PRO, patient‐reported outcome; SD, standard deviation.

^a^ AA disease severity (mild, moderate, or severe) was self‐reported by respondents and based on their own assessment of severity.

^b^ Current total area of scalp hair loss at the time of the survey.

^c^ Current AA treatment does not include phototherapy treatment.

^d^ Such as hats and head scarves.

^e^ Such as microblading on eyebrows.

^f^ Such as hair powders or fibers, arrangement of hair to cover bald spots, plastering hair down, and other hairstyles to conceal bald spots.

### 3.2 Treatments Used by Patients for AA and Satisfaction Level With Current Hair Growth

At the time of the survey, 8.1% (*n* = 38) of respondents were receiving prescription treatment, and 5.5% (*n* = 26) were receiving phototherapy for AA (Table 2). Among individuals receiving prescription treatment for AA, 81.6% (*n* = 31) and 36.8% (*n* = 14) were receiving topical treatment and oral treatment (including oral Janus kinase inhibitors, *n* = 2), respectively (Tables S2 and S3).

Additional approaches utilized by respondents to address their AA included headwear such as hats and scarves (9.8%, *n* = 46) and wigs (3.0%, *n* = 14) (Table 2). The proportions of respondents using headwear or wigs were higher for those with a severe case (37.5% and 29.2%, respectively) than those with mild (6.1% and 0.6%, respectively) or moderate (16.0% and 5.0%, respectively) cases.

**FIGURE 2:**
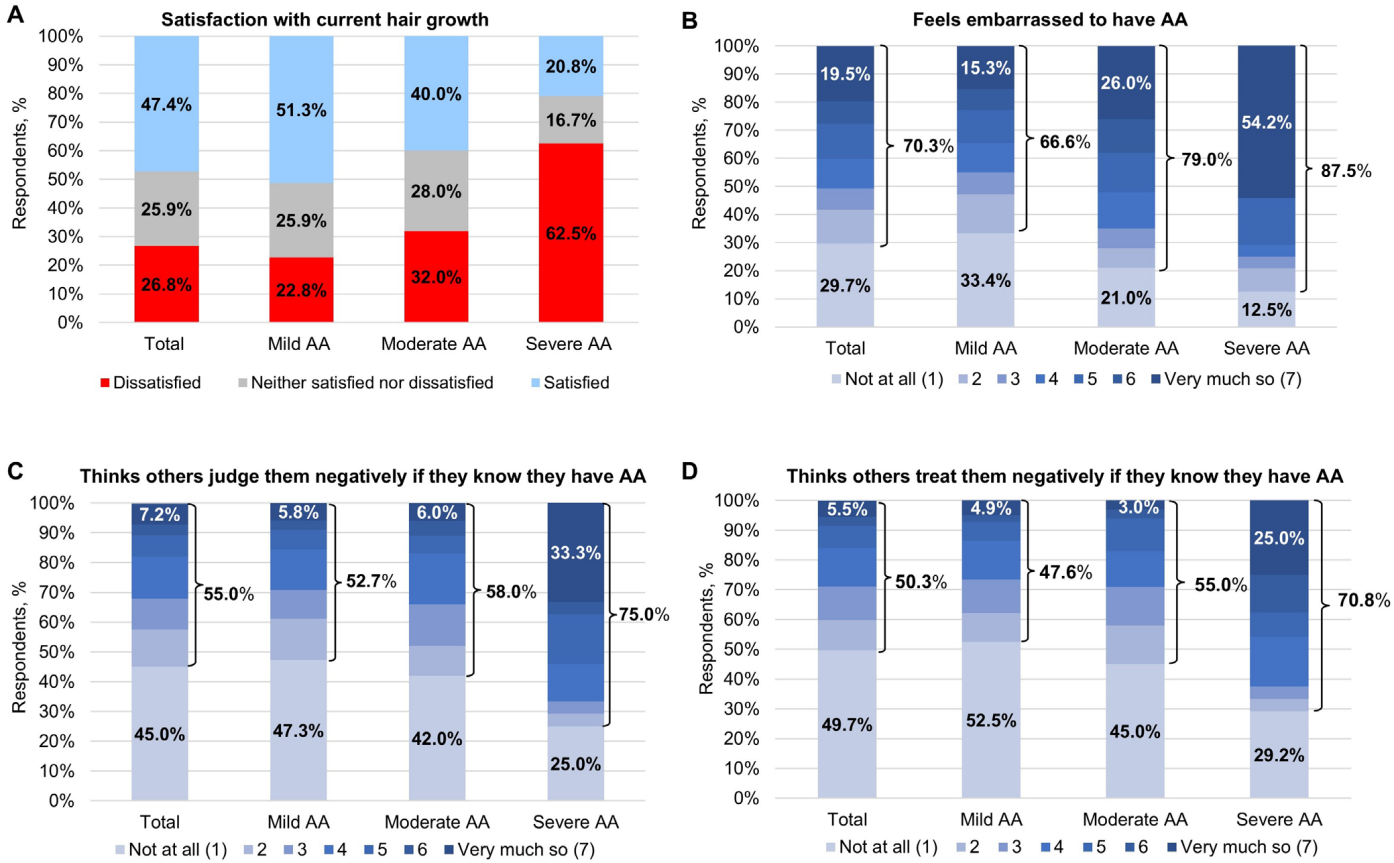
(A) Satisfaction with current hair growth among respondents and perceived stigma among respondents with (B) feelings of embarrassment, (C) negative judgment, and (D) being treated negatively due to AA by self‐reported disease severity^†^. AA, alopecia areata. ^†^AA disease severity (mild, moderate, or severe) was self‐reported by respondents and based on their own assessment of severity.

In total, 26.8% (*n* = 126) of respondents with diagnosed AA were dissatisfied with their current hair growth (Figure 2A). A higher proportion of respondents (62.5%) with a severe case were dissatisfied with their current hair growth than those reporting a mild (22.8%) or moderate (32.0%) case. Similar trends were observed when analyzing the data by respondents' current scalp hair loss range (Figure S1A).

### 3.3 Perceived Stigma Among Adults With AA

Overall, 70.3% of respondents reported feelings of embarrassment, 55.0% felt that others judged them negatively, and 50.3% felt that others treated them negatively due to AA (Figure 2B–D). More respondents (54.2%) with a severe case reported feeling “very much so” embarrassed to have AA than respondents who had a mild (15.3%) or moderate (26.0%) case (Figure 2B). Perceived AA‐associated stigma increased with disease severity, as higher proportions of respondents with a severe case reported feelings of AA‐related stigma than those with a mild or moderate case (Figure 2B–D). Additionally, perceived AA‐associated stigma generally increased with the extent of reported scalp hair loss (Figure S1B–D). However, among those with current scalp hair loss, the highest proportion who reported feeling “not at all” embarrassed to have AA were those who reported scalp hair loss of 95%–100% compared with other groups (30.8% vs. 9.1%–25.4%) (Figure S1B).

## 4 Discussion

The key finding of this study was the high level of perceived stigma experienced by adults with self‐reported AA in Japan. More than half of respondents felt embarrassed to have AA and felt that others judged them and treated them negatively because of their AA. Experiences of AA‐related stigma in Japan were also reported in a 2023 study that showed approximately half of adults with AA experienced negative effects on self‐esteem/self‐confidence and social interactions because of AA [7]. Mental health conditions may contribute to AA‐associated stigma. A systematic review examining the predictors and mechanisms of self‐stigma in patients with one of five chronic skin diseases, including AA, found that mental health and self‐stigma were significantly correlated in several studies [15]. Other factors that were reported to be associated with or predictive of self‐stigma among patients with chronic skin diseases included social stigma, coping strategies (including acceptance coping), sex, and disease severity and duration [15]. In this study, perceived AA‐associated stigma increased with disease severity and generally increased with the extent of scalp hair loss. Psychological stress is widely recognized as a triggering factor of AA [22]. After the appearance of scalp hair loss, patients may feel embarrassed about AA, as observed in this study, which may increase stress and potentially worsen their symptoms. Mental health support, interventions that target factors associated with AA‐related stigma, and treatment are essential for addressing both the psychological impact and the physical symptoms of AA for patients.

The self‐reported lifetime prevalence of AA among adults living in Japan was 3.9% in this study, with 1.6% of all adults reporting a physician diagnosis. These results are consistent with a 2023 study of the epidemiology of AA in Japan that reported a prevalence rate of approximately 1.45% [7]. A proportion of individuals in this study reported undiagnosed AA, showing that a subpopulation of individuals living in Japan has experienced AA but has not received a diagnosis from a physician. Individuals with patchy AA who have less extensive hair loss may cover bald patches with other hair, and spontaneous hair regrowth can occur in some cases [1, 23], preventing individuals from seeking out treatment or a diagnosis.

Severity of AA was self‐reported by respondents in this study. The proportion of respondents self‐reporting a mild case was lower than the proportion of those reporting scalp hair loss in the none to limited range (0%–20%; per the Alopecia Areata Investigator Global Assessment [AA‐IGA] scale) [24], while the proportion of respondents self‐reporting a moderate case was higher than those reporting scalp hair loss in the moderate range (21%–49%; per the AA‐IGA scale) [24]. These results indicate that patient experience of AA severity does not always align with the extent of scalp hair loss assessed by the SHA PRO. Hair loss across other body regions such as the face is not captured by the SHA PRO assessment and could affect patient perception of overall AA severity. Additionally, the impact of AA on activity levels, work productivity, and psychosocial health could contribute to patient perception of disease severity. A misalignment between patient‐reported disease severity and hair loss range was observed in a 2023 study of patients with AA in Japan, which showed that patients tended to rate their disease severity slightly lower than physicians who assessed severity by extent of scalp hair loss [13]. A 2022 study aiming to develop an AA severity scale demonstrated that along with extent of scalp hair loss, the psychosocial impact of AA, eyebrow/eyelash involvement, prior response to treatment, and rapid hair loss are important factors for determining AA severity [25]. The 2022 study developed the AA severity scale to capture all of these key features of AA [25]. Future studies could consider severity from various perspectives to take into consideration the patients' perspective and feelings, and the AA severity scale could be used when assessing patients with AA for disease severity.

Only 8.1% of respondents were currently receiving treatment for AA, which may have been due in part to the proportion of patients who reported 0% scalp hair loss at the time of the survey (29.3% of respondents) as well as the mean length of time since diagnosis. Many patients may have exhausted traditional treatments, which are associated with limited long‐term efficacy and safety. Less than half of respondents reported satisfaction with current hair growth, suggesting unmet treatment needs. With the approval of new treatments indicated for severe AA in Japan in 2022 and 2023, there should be an emphasis on increasing awareness of new treatment options among patients with AA [26].

In a previous 2022 Japanese study that examined health‐related quality of life in patients with AA, 46.0% were categorized as having anxiety (21.0%, doubtful anxiety; 25.0%, definite anxiety; 54.0%, no anxiety), and 41.8% were categorized as having depression (22.3%, doubtful depression; 19.5%, definite depression; 58.3%, no depression) [11], which is within the range of the globally estimated prevalence of mental health symptoms among patients with AA reported in a scoping review (30%–68%) [27]. However, in this study only 12% of all respondents with diagnosed AA reported experiencing a diagnosed mental health condition in the past year, suggesting that a subpopulation of individuals with AA in Japan has a mental health disorder that has not been diagnosed or treated. The proportions of respondents with diagnosed AA who had a diagnosed mental health disorder or sleep disorder were higher compared with the total sample of adults surveyed in this study, suggesting AA may impact mental health and sleep. More respondents with severe AA reported mental health conditions than those with mild or moderate disease, which is similar to other studies that have found an association between AA severity and mental health disorders, including anxiety and depression [10, 11, 13].

Several limitations should be considered when interpreting these results. NHWS data are self‐reported; consequently, recall error or other response bias may exist. Clinical verification of responses was not possible, and the number of severe AA cases is limited. Although the Japan NHWS is designed to reflect the demographic composition of the general adult population in Japan, it is possible that the data may not fully represent specific patient subpopulations examined in this study. For example, the respondents were enrolled in a self‐selected sample design, which may not reflect the entire population. However, when data were weighted based on age and sex to match the demographic composition of the adult population in Japan, similar results were obtained (data not shown).

In conclusion, adults living in Japan with AA reported considerable AA‐related stigma, mental health disorders, and dissatisfaction with current hair growth. These results highlight the psychological burden of AA and the importance of providing mental health care for patients with AA in Japan.

## Ethics Statement

Institutional review board approval was not required for this study; however, the protocols and materials associated with the 2023 Japan NHWS original fielding were reviewed by the Pearl Institute Review Board (Indianapolis, IN) and granted exemption from expedited or full ethical review. Respondents provided informed consent.

## Conflicts of Interest

N. Saito has received a speaker honorarium from Pfizer Japan Inc. K. Kamei and A. Yuasa are employees of Pfizer Japan Inc. and own stock and stock options in Pfizer Inc. G. Gautier, S.K. Kurosky, and G. Bell are employees of and own stock and/or stock options in Pfizer Inc. K.A. Hanson was a paid consultant for Pfizer during the period in which the work for this manuscript was conducted; the author is no longer affiliated with the company. L. Lee and N. Sternbach are employees of Oracle Life Sciences.

## Supporting information


Appendix S1.

## Data Availability

The data that support the findings of this study are available from Oracle Life Sciences, but restrictions apply to the availability of these data, which were used under license for the current study, and so are not publicly available.

## References

[1] C. H. Pratt , L. E. King, Jr. , A. G. Messenger , A. M. Christiano , and J. P. Sundberg , “Alopecia Areata,” Nature Reviews Disease Primers 3 (2017): 17011, 10.1038/nrdp.2017.11. PMC5573125 28300084

[2] M. A. Richard , F. Corgibet , M. Beylot‐Barry , et al., “Sex‐ and Age‐Adjusted Prevalence Estimates of Five Chronic Inflammatory Skin Diseases in France: Results of the << OBJECTIFS PEAU >> Study,” Journal of the European Academy of Dermatology and Venereology 32, no. 11 (2018): 1967–1971, 10.1111/jdv.14959. 29569785

[3] M. Harries , A. E. Macbeth , S. Holmes , et al., “The Epidemiology of Alopecia Areata: A Population‐Based Cohort Study in UK Primary Care,” British Journal of Dermatology 186, no. 2 (2022): 257–265, 10.1111/bjd.20628. 34227101 PMC9298423

[4] H. H. Lee , E. Gwillim , K. R. Patel , et al., “Epidemiology of Alopecia Areata, Ophiasis, Totalis, and Universalis: A Systematic Review and Meta‐Analysis,” Journal of the American Academy of Dermatology 82, no. 3 (2020): 675–682, 10.1016/j.jaad.2019.08.032. 31437543

[5] M. Benigno , K. P. Anastassopoulos , A. Mostaghimi , et al., “A Large Cross‐Sectional Survey Study of the Prevalence of Alopecia Areata in the United States,” Clinical, Cosmetic and Investigational Dermatology 13 (2020): 259–266, 10.2147/CCID.S245649. 32280257 PMC7131990

[6] A. C. Villasante Fricke and M. Miteva , “Epidemiology and Burden of Alopecia Areata: A Systematic Review,” Clinical, Cosmetic and Investigational Dermatology 8 (2015): 397–403, 10.2147/CCID.S53985. 26244028 PMC4521674

[7] T. Aranishi , T. Ito , M. Fukuyama , et al., “Prevalence of Alopecia Areata in Japan: Estimates From a Nationally Representative Sample,” Journal of Dermatology 50, no. 1 (2023): 26–36, 10.1111/1346-8138.16606. 36412271 PMC10100223

[8] E. Campos‐Alberto , T. Hirose , L. Napatalung , and M. Ohyama , “Prevalence, Comorbidities, and Treatment Patterns of Japanese Patients With Alopecia Areata: A Descriptive Study Using Japan Medical Data Center Claims Database,” Journal of Dermatology 50, no. 1 (2023): 37–45, 10.1111/1346-8138.16615. 36321512 PMC10092019

[9] F. Rencz , L. Gulácsi , M. Péntek , N. Wikonkál , P. Baji , and V. Brodszky , “Alopecia Areata and Health‐Related Quality of Life: A Systematic Review and Meta‐Analysis,” British Journal of Dermatology 175, no. 3 (2016): 561–571, 10.1111/bjd.14497. 26914830

[10] E. Edson‐Heredia , T. Aranishi , Y. Isaka , P. Anderson , S. Marwaha , and J. Piercy , “Patient and Physician Perspectives on Alopecia Areata: A Real‐World Assessment of Severity and Burden in Japan,” Journal of Dermatology 49, no. 6 (2022): 575–583, 10.1111/1346-8138.16360. 35343611 PMC9310764

[11] T. Ito , K. Kamei , A. Yuasa , et al., “Health‐Related Quality of Life in Patients With Alopecia Areata: Results of a Japanese Survey With Norm‐Based Comparisons,” Journal of Dermatology 49, no. 6 (2022): 584–593, 10.1111/1346-8138.16364. 35342979 PMC9314875

[12] N. Mesinkovska , B. King , P. Mirmirani , J. Ko , and J. Cassella , “Burden of Illness in Alopecia Areata: A Cross‐Sectional Online Survey Study,” Journal of Investigative Dermatology. Symposium Proceedings 20, no. 1 (2020): S62–S68, 10.1016/j.jisp.2020.05.007. 33099390

[13] K. Nakamura , K. Kamei , J. Austin , et al., “Degree of Alignment Between Japanese Patients and Physicians on Alopecia Areata Disease Severity and Treatment Satisfaction: A Real‐World Survey,” Dermatology and Therapy 14, no. 1 (2024): 151–167, 10.1007/s13555-023-01067-y. 38079099 PMC10828166

[14] M. Ohyama , K. Kamei , A. Yuasa , P. Anderson , G. Milligan , and M. Sakaki‐Yumoto , “Economic Burden of Alopecia Areata: A Study of Direct and Indirect Cost in Japan Using Real‐World Data,” Journal of Dermatology 50, no. 10 (2023): 1246–1254, 10.1111/1346-8138.16888. 37435720

[15] C. F. Z. Stuhlmann , J. Traxler , V. Paucke , N. da Silva Burger , and R. Sommer , “Predictors and Mechanisms of Self‐Stigma in Five Chronic Skin Diseases: A Systematic Review,” Journal of the European Academy of Dermatology and Venereology 39, no. 3 (2025): 622–630, 10.1111/jdv.20314. 39247975 PMC11851255

[16] Y. Higaki , I. Watanabe , T. Masaki , et al., “Japanese Version of Cutaneous Body Image Scale: Translation and Validation,” Journal of Dermatology 36, no. 9 (2009): 477–484, 10.1111/j.1346-8138.2009.00690.x. 19712274

[17] F. Matzer , J. W. Egger , and D. Kopera , “Psychosocial Stress and Coping in Alopecia Areata: A Questionnaire Survey and Qualitative Study Among 45 Patients,” Acta Dermato‐Venereologica 91, no. 3 (2011): 318–327, 10.2340/00015555-1031. 21290087

[18] A. B. Temel , S. Bozkurt , Y. Senol , and E. Alpsoy , “Internalized Stigma in Patients With Acne Vulgaris, Vitiligo, and Alopecia Areata,” Turkish Journal of Dermatology 13, no. 3 (2019): 109–116, 10.4103/tjd.Tjd_14_19.

[19] M. D. DiBonaventura , J. S. Wagner , Y. Yuan , G. L'Italien , P. Langley , and W. Ray Kim , “Humanistic and Economic Impacts of Hepatitis C Infection in the United States,” Journal of Medical Economics 13, no. 4 (2010): 709–718, 10.3111/13696998.2010.535576. 21091098

[20] K. W. Wyrwich , H. Kitchen , S. Knight , et al., “Development of the Scalp Hair Assessment PRO Measure for Alopecia Areata,” British Journal of Dermatology 183, no. 6 (2020): 1065–1072, 10.1111/bjd.19024. 32163589 PMC7754291

[21] United States Census Bureau , “International Database: World Population Estimates and Projections,” United States Census Bureau, October 15, 2024, https://www.census.gov/programs‐surveys/international‐programs/about/idb.html.

[22] Y. Minokawa , Y. Sawada , and M. Nakamura , “Lifestyle Factors Involved in the Pathogenesis of Alopecia Areata,” International Journal of Molecular Sciences 23, no. 3 (2022): 1038, 10.3390/ijms23031038. 35162962 PMC8835065

[23] F. Akritidou , K. Misiou , E. Tsanidou , G. N. Katsaras , and T. Papamitsou , “Unexpected Hair Regrowth in a Woman With Longstanding Alopecia Universalis,” GMS Ophthalmology Cases 11 (2021): Doc08, 10.3205/oc000181. PMC8167370 34123698

[24] K. W. Wyrwich , H. Kitchen , S. Knight , et al., “The Alopecia Areata Investigator Global Assessment Scale: A Measure for Evaluating Clinically Meaningful Success in Clinical Trials,” British Journal of Dermatology 183, no. 4 (2020): 702–709, 10.1111/bjd.18883. 31970750 PMC7586961

[25] B. A. King , N. A. Mesinkovska , B. Craiglow , et al., “Development of the Alopecia Areata Scale for Clinical Use: Results of an Academic‐Industry Collaborative Effort,” Journal of the American Academy of Dermatology 86, no. 2 (2022): 359–364, 10.1016/j.jaad.2021.08.043. 34474079

[26] Pharmaceuticals and Medical Devices Agency (PMDA) , “List of Approved Products,” accessed June 21 (2025), https://www.pmda.go.jp/english/review‐services/reviews/approved‐information/drugs/0002.html.

[27] A. Muntyanu , S. Gabrielli , J. Donovan , et al., “The Burden of Alopecia Areata: A Scoping Review Focusing on Quality of Life, Mental Health and Work Productivity,” Journal of the European Academy of Dermatology and Venereology 37 (2023): 1490–1520, 10.1111/jdv.18926. 36708097

