# Supplementary material for: Perceived Stigma and Mental Health Disorders Among Adults With Alopecia Areata Living in Japan
Source: J Dermatol. 2025 Jun 27;52(8):1255–62. doi: 10.1111/1346-8138.17831 (PMC12315609; doi:10.1111/1346-8138.17831)
Supplement: Supplementary file 1 — Appendix S1. [file JDE-52-1255-s001.docx]

# **SUPPLEMENTAL MATERIALS**

**Table S1.** Demographics, baseline characteristics, and clinical characteristics by self-reported current scalp hair loss range per the SHA PRO^TM^

| Characteristic | Current scalp hair loss range (PRO) | | | | |
| --- | --- | --- | --- | --- | --- |
|  | 0%  (n=138) | 1-20%  (n=280) | 21-49%  (n=29) | 50-94%  (n=11) | 95-100%  (n=13) |
| Age, mean (SD) | 56.4 (14.9) | 56.3 (15.6) | 58.4 (12.6) | 56.1 (14.1) | 56.2 (9.1) |
| Sex, n (%) | | | | | |
| Male | 47 (34.1) | 111 (39.6) | 21 (72.4) | 4 (36.4) | 6 (46.2) |
| Female | 91 (65.9) | 169 (60.4) | 8 (27.6) | 7 (63.6) | 7 (53.9) |
| Employment status, n (%) | | | | | |
| Full time, part time, or self-employed | 68 (49.3) | 147 (52.5) | 21 (72.4) | 4 (36.4) | 10 (76.9) |
| Unemployed^†^ | 70 (50.7) | 133 (47.5) | 8 (27.6) | 7 (63.6) | 3 (23.1) |
| Insurance coverage, n (%) | | | | | |
| National health insurance | 68 (49.3) | 135 (48.2) | 11 (37.9) | 3 (27.3) | 4 (30.8) |
| Social insurance | 57 (41.3) | 111 (39.6) | 12 (41.4) | 4 (36.4) | 8 (61.5) |
| Late-stage elderly insurance | 6 (4.4) | 21 (7.5) | 3 (10.3) | 2 (18.2) | - |
| Other | 3 (2.2) | 12 (4.3) | 2 (6.9) | - | - |
| None of the above | 4 (2.9) | 1 (0.4) | 1 (3.5) | 2 (18.2) | 1 (7.7) |
| Diagnosed comorbidities, n (%) | | | | | |
| Atopic dermatitis (in the past 12 months) | 9 (6.5) | 26 (9.3) | 6 (20.7) | 2 (18.2) | 3 (23.1) |
| Thyroid condition (ever) | 9 (6.5) | 19 (6.8) | 2 (6.9) | 1 (9.1) | 1 (7.7) |
| Psoriasis (ever) | 4 (2.9) | 12 (4.3) | 1 (3.5) | - | 1 (7.7) |
| Rheumatoid arthritis (ever) | 3 (2.2) | 11 (3.9) | - | - | 1 (7.7) |
| Vitiligo (ever) | 1 (0.7) | 1 (0.4) | - | - | 1 (7.7) |
| Psoriatic arthritis (ever) | - | 4 (1.4) | - | - | 1 (7.7) |
| Type 1 diabetes (ever) | 1 (0.7) | - | - | - | 1 (7.7) |
| Emotional or mental health conditions in the past 12 months^‡^ | 14 (10.1) | 34 (12.1) | 5 (17.2) | - | 4 (30.8) |
| Depression | 7 (5.1) | 28 (10.0) | 3 (10.3) | - | 3 (23.1) |
| Panic disorder | 5 (3.6) | 8 (2.9) | 3 (10.3) | - | 2 (15.4) |
| Anxiety | 5 (3.6) | 7 (2.5) | 1 (3.5) | - | 1 (7.7) |
| Sleep conditions in the past 12 months^§^ | 18 (13.0) | 40 (14.3) | 7 (24.1) | 1 (9.1) | 1 (7.7) |
| Insomnia | 17 (12.3) | 30 (10.7) | 6 (20.7) | 1 (9.1) | 1 (7.7) |
| Sleep apnea | - | 10 (3.6) | 2 (6.9) | - | 1 (7.7) |
| Narcolepsy | - | 1 (0.4) | - | - | 1 (7.7) |
| Sleep difficulties^‖^ | 1 (0.7) | 4 (1.4) | - | - | 1 (7.7) |
| Diagnosing physician for AA, n (%) | | | | | |
| Dermatologist | 109 (79.0) | 219 (78.2) | 20 (69.0) | 7 (63.6) | 8 (61.5) |
| General internist | 18 (13.0) | 32 (11.4) | 5 (17.2) | 1 (9.1) | 5 (38.5) |
| Other | 9 (6.5) | 21 (7.5) | 3 (10.3) | 2 (18.2) | - |
| Pediatrician | 1 (0.7) | 6 (2.1) | - | 1 (9.1) | - |
| Unknown/not reported | 1 (0.7) | 2 (0.7) | 1 (3.5) | - | - |
| Time since first diagnosis, mean (SD), years | 23.1 (16.3) | 19.9 (16.8) | 17.9 (13.8) | 24.8 (18.4) | 35.7 (17.8) |
| Severity, n (%) | | | | | |
| Mild | 118 (85.5) | 214 (76.4) | 13 (44.8) | 1 (9.1) | 1 (7.7) |
| Moderate | 18 (13.0) | 61 (21.8) | 14 (48.3) | 3 (27.3) | 4 (30.8) |
| Severe | 2 (1.5) | 5 (1.8) | 2 (6.9) | 7 (63.6) | 8 (61.5) |
| Currently receiving treatment for AA^¶^, n (%) | | | | | |
| Yes | 2 (1.5) | 30 (10.7) | 5 (17.2) | - | 1 (7.7) |
| Currently receiving phototherapy, n (%) | | | | | |
| Yes | 4 (2.9) | 17 (6.1) | 3 (10.3) | 1 (9.1) | 1 (7.7) |
| Approaches currently using to address AA, n (%) | | | | | |
| Headwear^#^ | 3 (2.2) | 30 (10.7) | 8 (27.6) | 3 (27.3) | 2 (15.4) |
| Wigs | - | 6 (2.1) | 2 (6.9) | 2 (18.2) | 4 (30.8) |
| Tattoos mimicking the^††^ appearance of hair | - | - | 1 (3.5) | - | 1 (7.7) |
| Hair styling practices^‡‡^ | 1 (0.7) | 15 (5.4) | 2 (6.9) | - | 1 (7.7) |
| Psychological counseling for anxiety, depression, or sleep disorder related to AA | - | 3 (1.1) | 1 (3.5) | - | 1 (7.7) |
| Prescribed medication for treatment of anxiety, depression, or sleep disorder related to AA | 1 (0.7) | 4 (1.4) | 1 (3.5) | - | 1 (7.7) |
| Other | - | 7 (2.5) | - | 1 (9.1) | 1 (7.7) |
| None of the above | 133 (96.4) | 228 (81.4) | 20 (69.0) | 8 (72.7) | 8 (61.5) |

AA, alopecia areata, SD, standard deviation; SHA PRO, Scalp Hair Assessment patient-reported outcome.

^†^Includes homemaker, retired, student, short- or long-term leave of absence due to illness, not employed but looking for work, and not employed and not looking for work.

^‡^Other selected conditions included attention deficit/deficit and hyperactivity disorder, generalized anxiety disorder, obsessive compulsive disorder, phobias, post-traumatic stress disorder, and social anxiety disorder. Anxiety, depression, and panic disorder are not mutually exclusive.

^§^Other selected conditions included idiopathic hypersomnia.

^‖^Other than insomnia, narcolepsy, or sleep apnea.

^¶^Current AA treatment does not include phototherapy treatment.

^#^Such as hats and head scarves.

^††^Such as microblading on eyebrows.

^‡‡^Such as hair powders or fibers, arrangement of hair to cover bald spots, plastering hair down, and other hair styles to conceal bald spots.

**Table S2.** Current AA treatments utilized by respondents who reported treatment^†^

| Characteristic, n (%) | Diagnosed with AA | | | |
| --- | --- | --- | --- | --- |
|  | Total  (N=38) | Mild^‡^  (n=24) | Moderate^‡^  (n=10) | Severe^‡^  (n=4) |
| Topical^§^ | 31 (81.6) | 19 (79.2) | 8 (80.0) | 4 (100.0) |
| Oral | 14 (36.8) | 7 (29.2) | 4 (40.0) | 3 (75.0) |
| Injection at areas of hair loss | 4 (10.5) | 2 (8.3) | - | 2 (50.0) |
| Other injectable | 2 (5.3) | - | - | 2 (50.0) |
| Other | 6 (15.8) | 3 (12.5) | 2 (20.0) | 1 (25.0) |
| Unknown | 4 (10.5) | 2 (8.3) | 2 (20.0) | - |
| None listed | 2 (5.3) | 2 (8.3) | - | - |

AA, alopecia areata.

^†^Current AA treatment routes of administration are not mutually exclusive.

^‡^AA disease severity (mild, moderate, or severe) was self-reported by respondents and based on their own assessment of severity.

^§^Topical therapy included corticosteroids and other treatments.

**Table S3.** Current oral prescription treatments utilized by respondents who reported oral treatment^†^

| Characteristic, n (%) | Diagnosed with AA | | | |
| --- | --- | --- | --- | --- |
|  | Total  (N=14) | Mild^‡^  (n=7) | Moderate^‡^  (n=4) | Severe^‡^  (n=3) |
| Oral antihistamines | 7 (50.0) | 2 (28.6) | 2 (50.0) | 3 (100.0) |
| Oral corticosteroids | 5 (35.7) | 3 (42.9) | - | 2 (66.7) |
| Oral immunosuppressants | 3 (21.4) | 1 (14.3) | - | 2 (66.7) |
| Oral JAK inhibitors | 2 (14.3) | - | - | 2 (66.7) |
| Oral minoxidil | 1 (7.1) | - | - | 1 (33.3) |
| Oral other | 6 (42.9) | 2 (28.6) | 1 (25.0) | 3 (100.0) |
| Unknown^§^ | 2 (14.3) | 1 (14.3) | 1 (25.0) | - |

AA, alopecia areata; JAK, Janus kinase.

^†^Current oral treatments are not mutually exclusive.

^‡^AA disease severity (mild, moderate, or severe) was self-reported by respondents and based on their own assessment of severity.

^§^Patient selected taking oral therapy but did not select any oral medications.

**Figure S1. (A)** Satisfaction with current hair growth among respondents and perceived stigma among respondents with (**B**) feelings of embarrassment, (**C**) negative judgment, and (**D**) being treated negatively due to AA by self-reported current scalp hair loss range per the SHA PRO^TM^


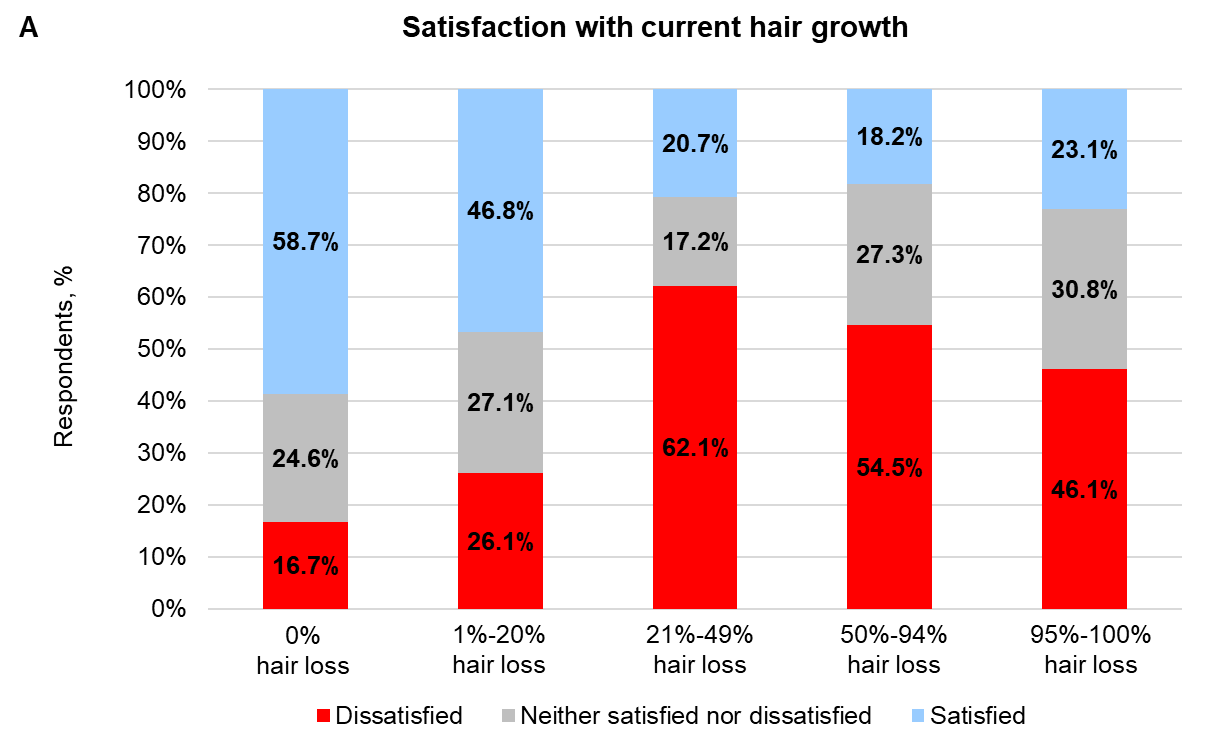


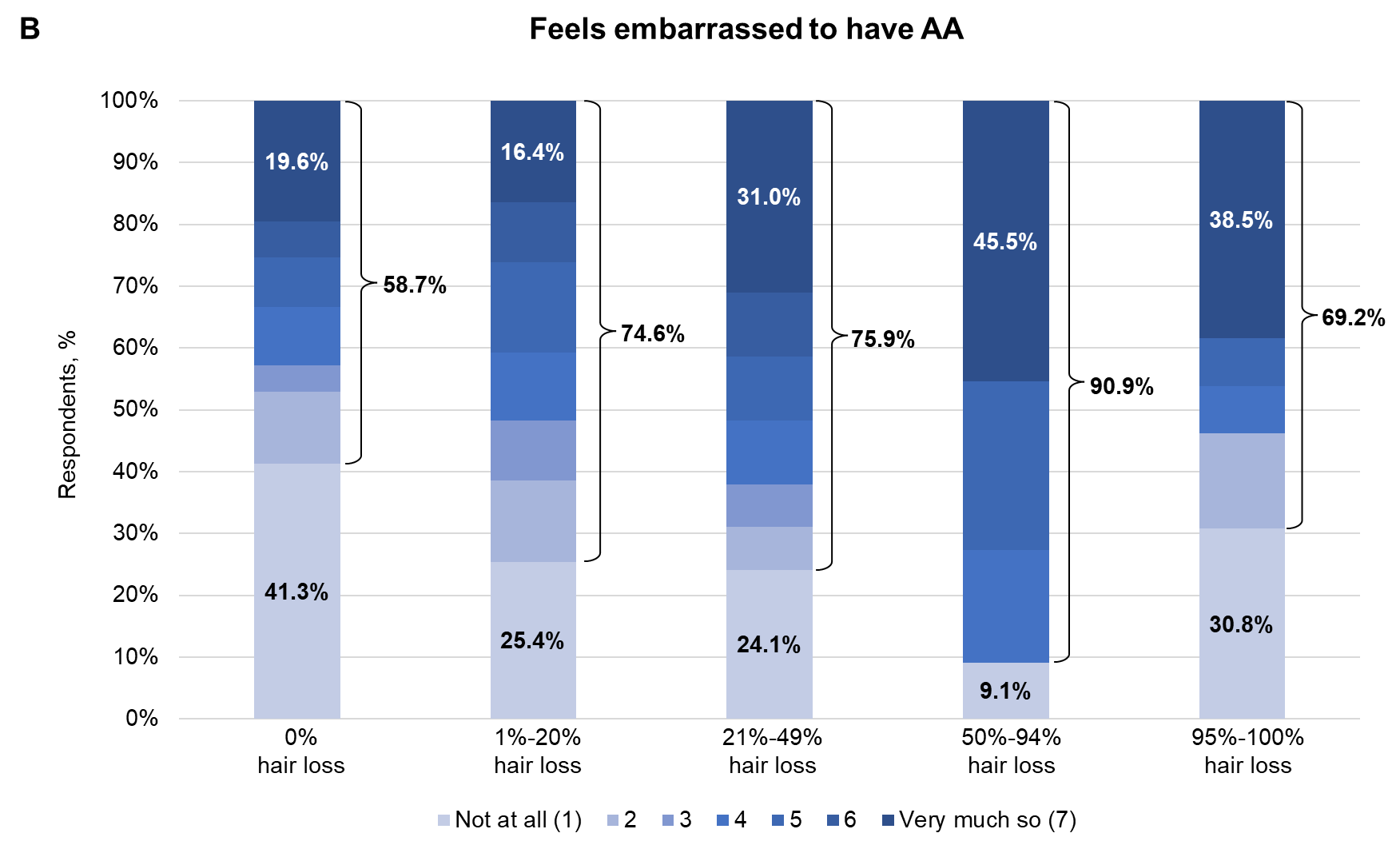


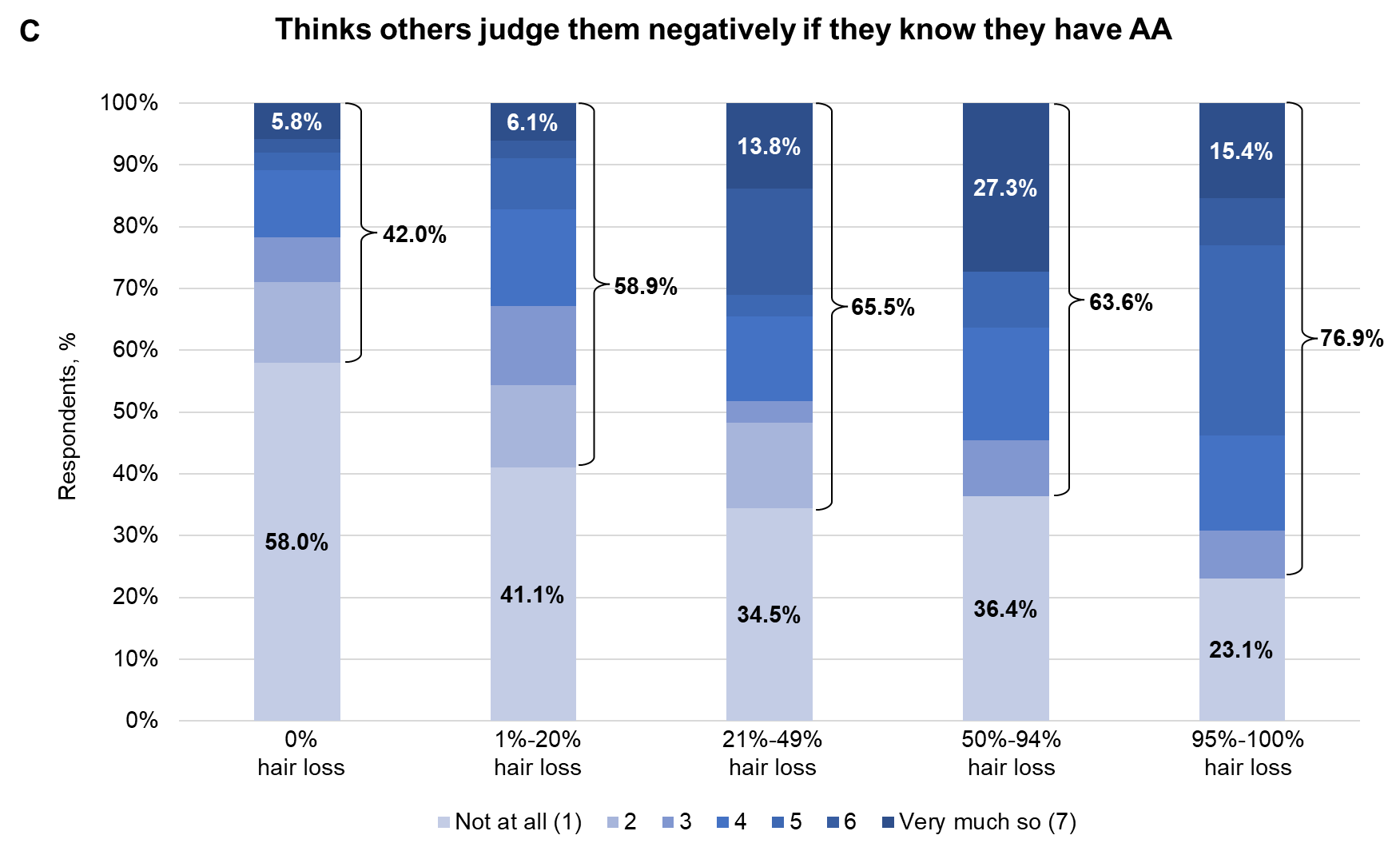


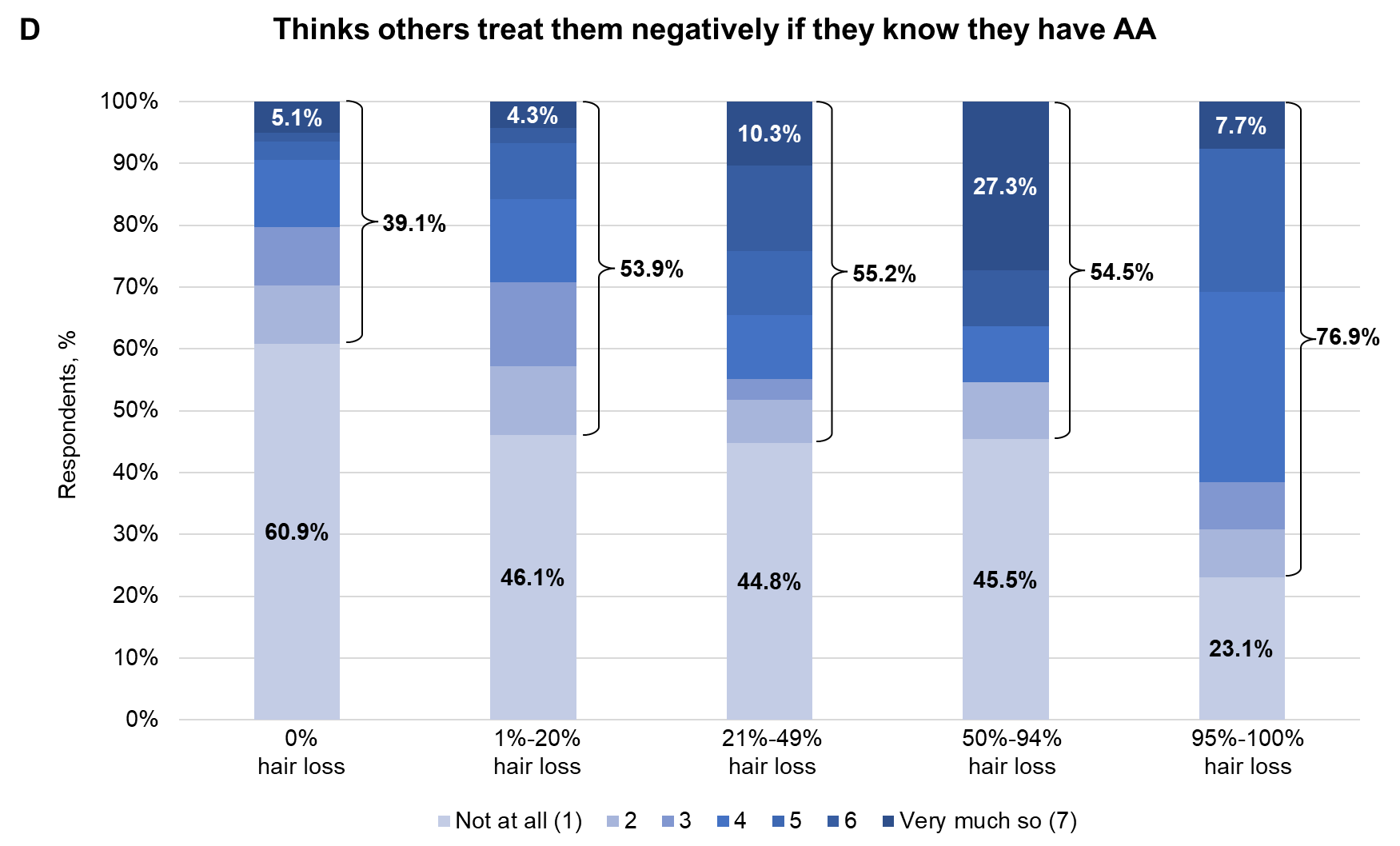


AA, alopecia areata; SHA PRO, Scalp Hair Assessment patient-reported outcome.
